# Suppressing Side-Lobe Radiations of Horn Antenna by Loading Metamaterial Lens

**DOI:** 10.1038/srep09113

**Published:** 2015-03-13

**Authors:** Mei Qing Qi, Wen Xuan Tang, Hui Feng Ma, Bai Cao Pan, Zui Tao, Yong Zhi Sun, Tie Jun Cui

**Affiliations:** 1 State Key Laboratory of Millimeter Waves, Southeast University, Nanjing 210096, China; 2 Synergetic Innovation Center of Wireless Communication Technology, Southeast University, Nanjing 210096, China

## Abstract

We propose a new approach to control the amplitude and phase distributions of electromagnetic fields over the aperture of a horn antenna. By loading a metamaterial lens inside the horn antenna, a tapered amplitude distribution of the aperture field is achieved, which can suppress the side-lobe radiations of the antenna. The metamaterial is further manipulated to achieve a flat phase distribution on the horn aperture to avoid the gain reduction that usually suffers in the conventional low-sidelobe antenna designs. A prototype of the metamaterial-loaded horn antenna is designed and fabricated. Both numerical simulations and measured results demonstrate the tapered aperture-field distribution and significant reduction of side-lobe and back-lobe radiations in the operating frequency band.

In the past decade, metamaterials composed of periodic structures in the sub-wavelength scales have provided an unprecedented skill to control electromagnetic waves and create new devices such as invisibility cloaks^1^,^2^,^3^,^4^,^5^, perfect absorbers^6^,^7^, metamaterial lenses^8^,^9^,^10^,^11^,^12^,^13^,^14^,^15^,^16^, and metamaterial antennas^17^,^18^,^19^,^20^,^21^. Among various metamaterials in literatures, the gradient refractive index (GRIN) metamaterials which possess gradient permittivity and/or permeability have played an important role. The GRIN metamaterials can be artificially realized with different unit cells with varying geometries and dimensions, and therefore, is simple to design. The first GRIN metamaterial was implemented in 2005, consisting of split-ring resonators (SRRs) to form the deflection of microwave beam^8^. Since then, the GRIN metamaterials have been utilized in achieving a variety of microwave applications, such as the Luneburg lenses^11^,^12^, highly directive antennas^13^,^14^, Maxwell fish-eye lenses^15^,^16^, low sidelobe lens antennas^17^, and beam-scanning antenna^10^. In most literatures, the GRIN metamaterials have served to increase the directivity and gain of antennas by transforming spherical waves to plane waves^11^,^12^,^13^,^14^,^15^,^16^.

More specifically, an efficient method was proposed in Ref. 17 to control both the amplitude and phase distributions of the metamaterial lens, in which the spherical waves emitted from a special source (i.e., a well-designed waveguide antenna) are converted to plane waves, achieving a performance-improved lens antenna^17^. However, this metamaterial lens has to be placed away from the specially designed source so that the far-field radiation patterns of the source can be used to illuminate the surface of the metamaterial lens. More recently, Gok and Grbic presented a method to tailor the electromagnetic fields including the phase and power flow^18^. But the required inhomogeneous and anisotropic medium is hard to be implemented.

In this article, we propose a new approach of manipulating the aperture field and succeed to reduce the side-lobe level of radiation patterns by loading GRIN metamaterials in a three-dimensional (3D) horn antenna. It is well known that the horn antennas have been widely used in wireless communication and satellite communication systems, due to their simple structures, broad operating bandwidth, low loss, and high reliability. However, the horn antennas have also some intrinsic drawbacks, such as the non-ideal aperture field distribution, huge discrepancy of radiation patterns in broad band, and the relatively high E-plane sidelobes^19^,^20^. The metamaterials adopted here are placed inside the horn antenna without increasing its size, and can manipulate both amplitude and phase distributions of the aperture fields, and consequently control the property of horn-antenna radiations.

## Results

### Mechanism of manipulating aperture fields

The basic theory of the GRIN metamaterial has been performed in Ref. 8. It has been shown that the wavefront can be manipulated by a GRIN lens. Here, we will demonstrate further that the GRIN metamaterials can not only be used to transform a spherical wave to a plane wave, they can also be employed to manipulate the distribution of the aperture field including magnitude and phase simultaneously.

**Figure 1:**
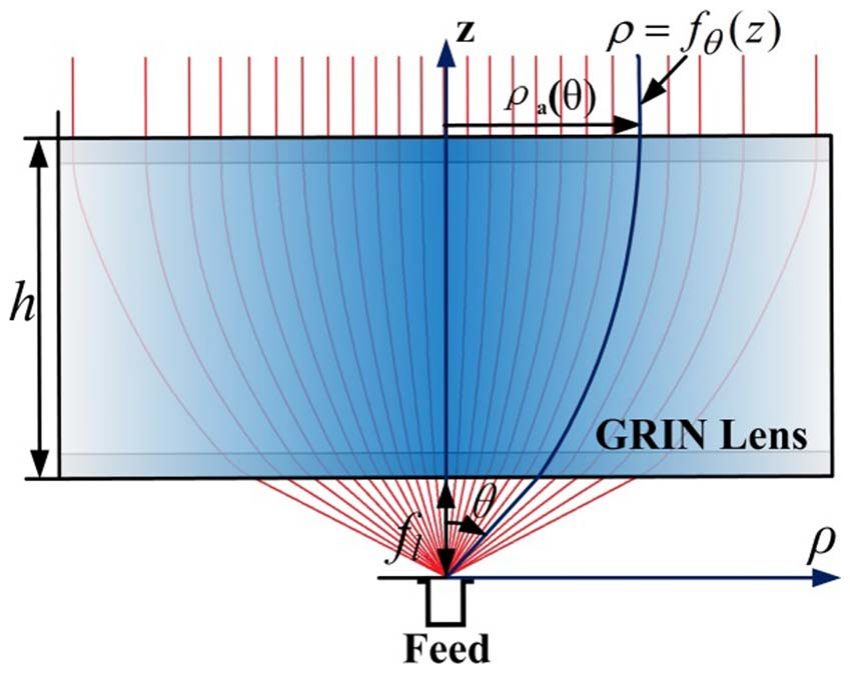
The schematic diagram of manipulating aperture field using GRIN lens.

In general, the distribution of refractive index of an arbitrarily-shaped lens determines the amplitude and the phase of aperture field when the feed source is given. Hence we can manipulate the refractive index inside the lens to control the aperture field. Without losing generality, we consider a planar lens as an example shown in Fig. 1, whose refractive index *n* (*ρ*) is supposed to be graded along the transverse direction (*ρ* direction) of the propagating wave. If it is required to achieve a highly-directive beam in the direction normal to the flat lens, a uniform phase is needed on the flat aperture. Hence the spherical waves excited by a feeding source must be transformed to plane waves when propagating through the flat lens. According to the criterion that every optical path from the feed source to the upper surface of the GRIN lens should have the same phase delay, the distribution of refraction index along the radius has been demonstrated approximately in following form:^10^
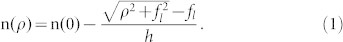


The technique of ray tracings is employed to indicate the propagating direction of the wave and to show the spherical-to-plane wave transformation (shown in Fig. 1). To demonstrate the ray tracings in a GRIN lens, the law of refraction is used for analysis. The refractive law can be used to calculate the deflection of a ray at the interface between different media. *n_i_* represents the refractive index at the point of (*ρ_i_, z_i_*) and *n_i_*_*+* *1*_ at the point of (*ρ_i_*_*+* *1*_*, z_i_*_*+* *1*_). According to the law of refraction, the iteration formation of the tangent of the ray tracing can be deduced as following:^17^


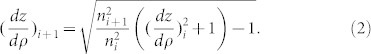
Hence, the ray tracing can be approximately discretized with short line segments. We can obtain the phase distribution *ϕ_a_* (*ρ*) of the field on the aperture by calculating the optical path (defined as 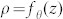)
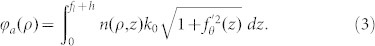
The radiation power of the feeding source is noted as *P_f_* (*θ*), and the power distribution on the lens aperture is noted as *P_a_*(*ρ*). According to the energy conservation law, we can obtain
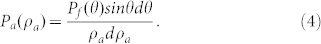


It is obviously shown that the refraction index of the lens determines the distribution of the aperture field. Therefore, we can manipulate the distribution of the aperture field over the aperture of the GRIN lens through tailoring the refractive index distribution.

### Design of the metamaterial-loaded horn antenna

**Figure 2:**
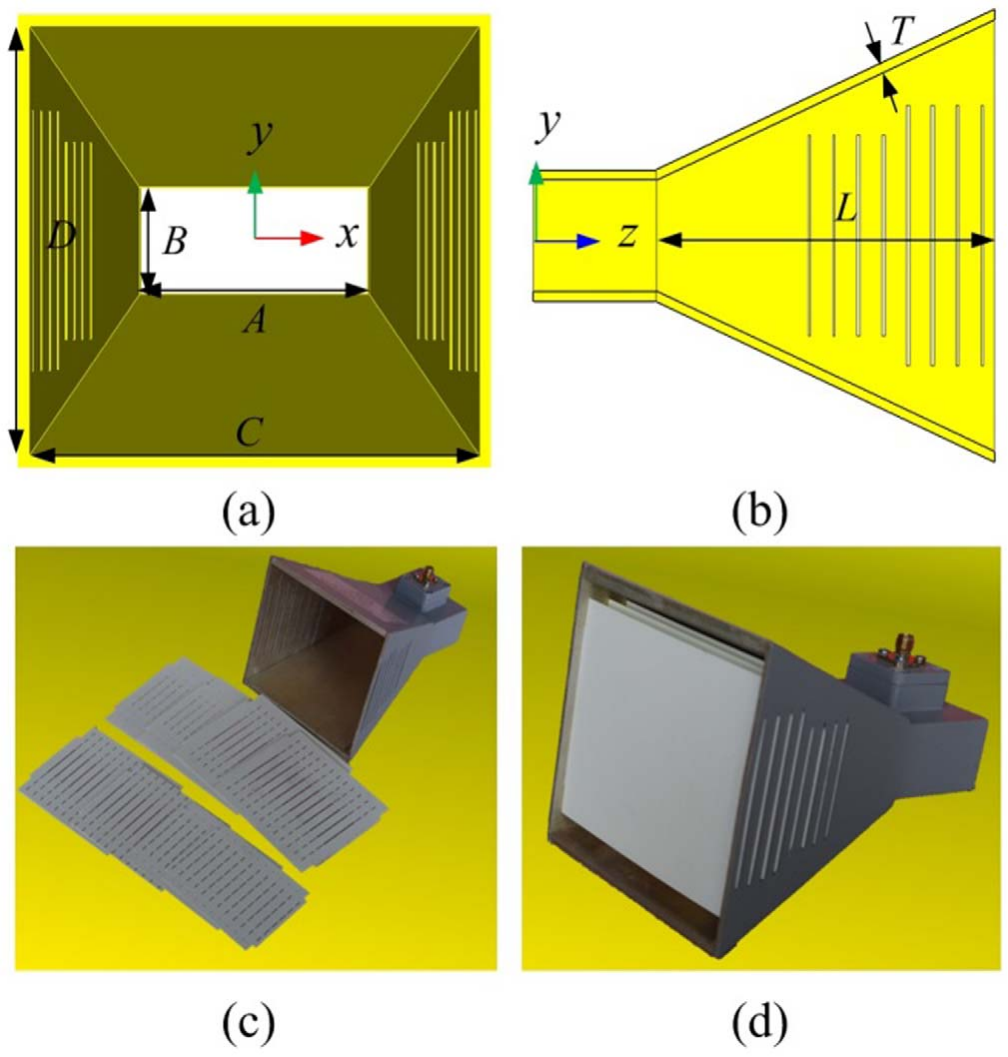
The topology of the proposed antenna. (a)–(b) The geometry of the proposed antenna. (c) The air horn (an empty horn filled with the air) and the comprising metamaterials. (d) The photograph of the meta-horn. The detailed dimensions (unit: mm) are: *A* = 47.55, *B* = 22.15, *C* = 90, *D* = 86, L = 82, and *T* = 4.

The performance of a pyramidal horn antenna can be improved using the proposed approach of manipulating aperture field. By loading a GRIN metamaterial lens inside a pyramidal horn, we succeed to suppress the side lobes of the far-field radiation patterns. Figure 2 illustrates the geometry and the photograph of the metamaterial- loaded antenna. Notice that equivalent media are used to replace metamaterials for design, since the theory of the equivalent theory of the metamaterial is able to efficiently simplify the process^22^. The metamaterial lens consists of a core layer (CL) and two impedance matching layers (IMLs). Following, the design of the loaded GRIN lens using equivalent media and its physical implementation are demonstrated in detail.

It is well known that the radiation patterns of an antenna are determined by its aperture field. In the traditional horn, the amplitude of tangential component of the aperture field is approximated by following forms:^23^
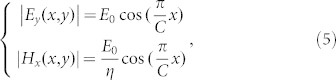
where C is aperture dimension of the pyramidal horn along x direction (see Fig. 2(a)).

**Figure 3:**
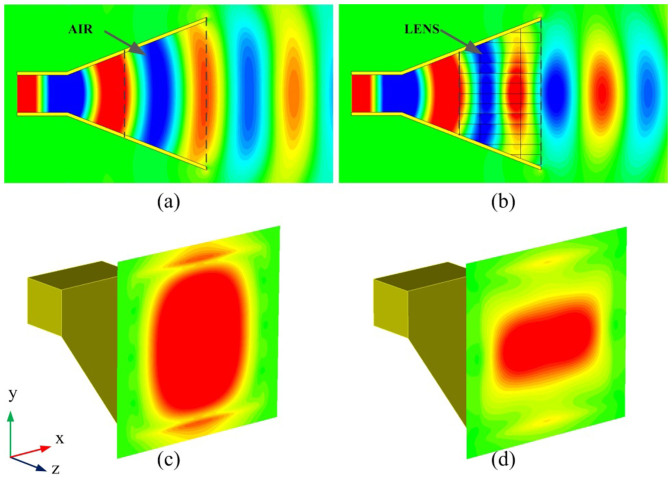
Electric field in E-plane of horns and amplitude of the electric field on the aperture. (a) (c) The air horn, (b) (d) The horn loaded with equivalent media.

**Figure 4:**
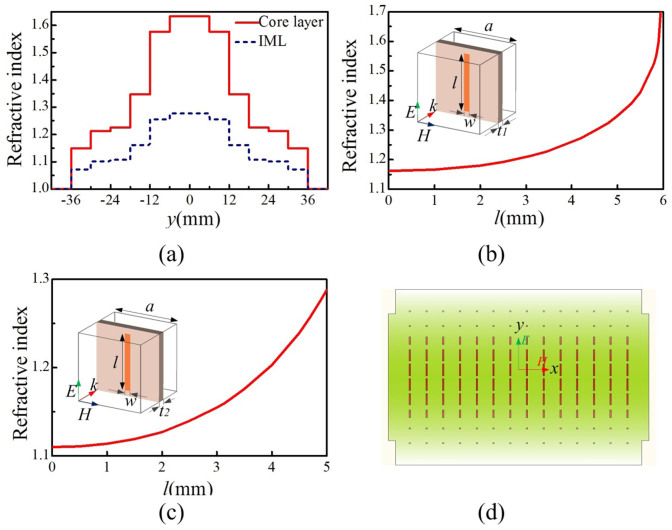
The refractive-index distribution and the realization method. (a) The refractive-index distribution along E-direction for the core layer and the impedance matching layers (IMLs). (b) The relation between length (*l*) of the metallic strip and the refractive-index (*n*) of metamaterials for the core layer. (c) The relation between length (*l*) of the metallic strip and the refractive-index (*n*) of metamaterials for the IMLs. (d) The schematic graph of the metallic strips. The detailed dimensions are: a = 6, w = 0.5, t1 = 0.762, t2 = 0.508 (unit: mm).

From Equation 5, we can obtain that the E-field on the aperture of an air horn (the empty pyramid horn) is nearly uniform in E-direction (y direction) and tapered in H-direction (x direction), as simulated in Fig. 3(a) or (c). This results in narrow beamwidth and high side-lobes in the E-plane. In order to reduce the side-lobe level in the radiation pattern, tapered distribution in E-direction is also required. GRIN metamaterial lens are adopted to the horn to modify the aperture field distribution in E-direction, making the amplitude distribution tapered and the phase distribution uniform. Through optimizing using initial value given in Eq. (1)^13^, the target refractive index distribution along y-direction (E-direction) of the GRIN metamaterial is achieved in Fig. 4(a). For comparison, electric fields on the aperture when metamaterials are loaded inside the horn (termed as the “meta-horn” here) are also shown in Fig. 3(b) and (d). In Fig. 3(b) the horn is filled with ideal metamaterial blocks that possessing well-designed refractive index distribution shown in Fig. 4(a). According to the law of refraction, it can be obtained that rays will bend when propagating through an inhomogeneous material^17^. Therefore, the GRIN metamaterial lens loaded on the apterture of the horn well manipulate the aperture field distribution and the electromagnetic energy to some extent be gathered to the central region of the aperture. Therefore, a tapered field distribution is formed on the aperture (see Fig. 3 (b) and (d)). It is observed that the distribution of the electric field in E-direction is also a tapered one. A tapered aperture field distribution is the key to suppress the side-lobes of the far-field radiation patterns.

In addition, as impedance mismatching happens on the interface between the GRIN metamaterial lens and the air, impedance matching layers (IMLs) are added to the GRIN metamaterial (which is termed as “the core layer” in the following text)^17^. According to the theory, the thickness of the IML is one quarter of the wavelength at the operating frequency. In practice, because the IML in broadband and the wavelength varies in different media, the thickness of the IML is approximately one-quarter of the wavelength at center frequency. In order to reduce the complexity for design and fabrication, permeabilities of the composing MTMs are restricted to be unity and the refractive index distribution for the IML is therefore decided by the square root of the refractive index in the core layer.

**Figure 5:**
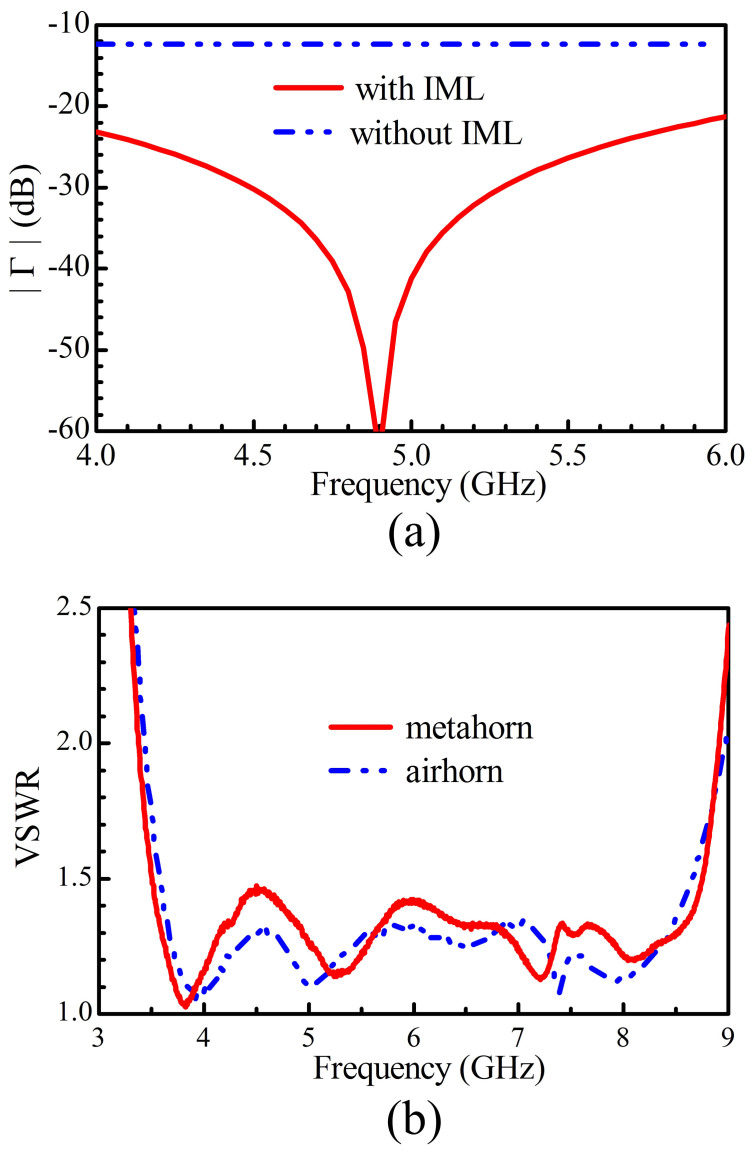
Matching performance. (a) The reflection in the central area on the interface between the lens and the air with and without the IMLs (*n_c_* = 1.278, *n_m_* = 1.633). (b) The measured VSWR.

In this work, the thickness for each IML (noted as *h_m_*) is chosen as two tenth of the wavelength (*λ_0_*) at the center frequency (*f_0_*), whose optical path is close *λ_0_*/4. The refractive index of the core layer and the IML are noted as *n_c_* and *n_m_*, respectively. The input impedance on the interface between the air and the IML is
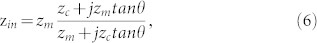
in which *z_m_* = 1/*n_m_*, *z_c_* = 1/*n_c_* and *θ* is the propagation phase in the IML. Therefore,
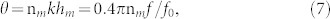
in which *f* is the operating frequency. Therefore, we can obtain the reflection coefficient at the interface between the lens and the air as:
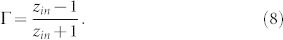


According to the distribution of the refractive index in Fig. 4(a) and the field distribution of the horn antenna, the main reflection occurs in the central area on the aperture if the IML is not applied. Figure 5 (a) presents the reflection on the lens aperture for the normal incidence with and without IML. It is clear that the reduction of the reflection is more than 10 dB over the entire operating frequency band when the IML is applied. Therefore, the IML is essential to improve the horn antenna performance in broadband.

### Realization of the GRIN lens using metamaterials

To implement the proposed GRIN lens, metamaterials with refractive index covering the range are needed. Metallic strips with different lengths are printed on the substrate of Rogers RO4350B to construct metamaterial lens. Relation between length of the strip and the refractive index of the metamaterial is illustrated in Figs. 4 (b) and (c). It is noted that in figure 4(b) the thickness (*t_1_*) of substrate is chosen to be 0.762 mm for the core layers, and in figure (c) the thickness (*t_2_*) is chosen to be 0.508 mm for the IMLs. For the substrate with thickness *t_1_*, the refractive index varies from 1.16 to 1.7 when the length of the strip changes from zero to 5.9 mm. While for the substrate with thickness *t_2_*, the refractive index varies from 1.11 to 1.29 corresponding to the length of the strip increasing from zero to 5 mm. Therefore, the refractive index can cover the target refractive index distribution.

Finally, the metamaterial-loaded antenna is composed of two parts – one is the pyramid horn with 16 parallel narrow slots cut along the walls (as shown in Fig. 2(b)), and the other is 8 pieces of metamaterial slices with different scales (as shown in Fig. 2(c)). The slices are made of Rogers RO4350B with printed metallic strips on it, and constructed inside the pyramid horn as shown in Fig 2(d). The core layer includes 4 slices and the IMLs include 4 slices. The shape of each slice is designed to evenly match the horn, as illustrated in Fig. 4(d) for example. The separation between the slices is 6 mm, which is one tenth of the wavelength at the center frequency of *f_0_* = 5 GHz.

## Discussion

**Figure 6:**
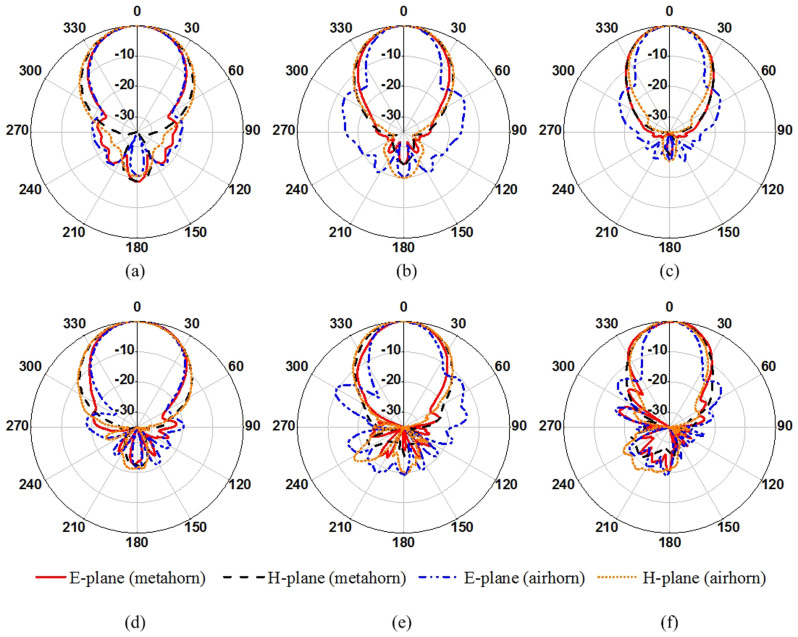
Simulated (a–c) and measured (d–f) far-field radiation patterns of the air horn and the meta-horn at 4, 5 and 6 GHz, respectively.

We have performed numerical simulation for the proposed meta-horn antenna and air horn in CST microwave studio. The simulated far-field radiation patterns of the air horn and the meta-horn with real structures of metallic strips at three sample frequencies (4, 5 and 6 GHz) are shown in Figs. 6(a)–(c). It is observed that for the air horn the beam-width in the E-plane is narrower than that in the H-plane and the side-lobes in the E-plane is very high. This is in good agreement with the analysis of distribution of the aperture field. In contrast, for the meta-horn the performance is enhanced obviously. It has apparently lower side-lobes compared with the air horn, proving that one can effectively reduce the side-lobe of a horn antenna by loading metamaterials; its beam-width in the E-plane is broader than that for the air horn, especially at higher frequencies; it has identical beam-widths in the E-plane and the H-plane at 6 GHz. In addition, it is also noted that the beam-widths in the H-plane are almost the same for the air horn and the meta-horn, which is due to the fact that gradient refractive index is only distributed in E-direction. The measured results agree well with the simulated ones, as plotted in Fig. 6 as well.

**Figure 7:**
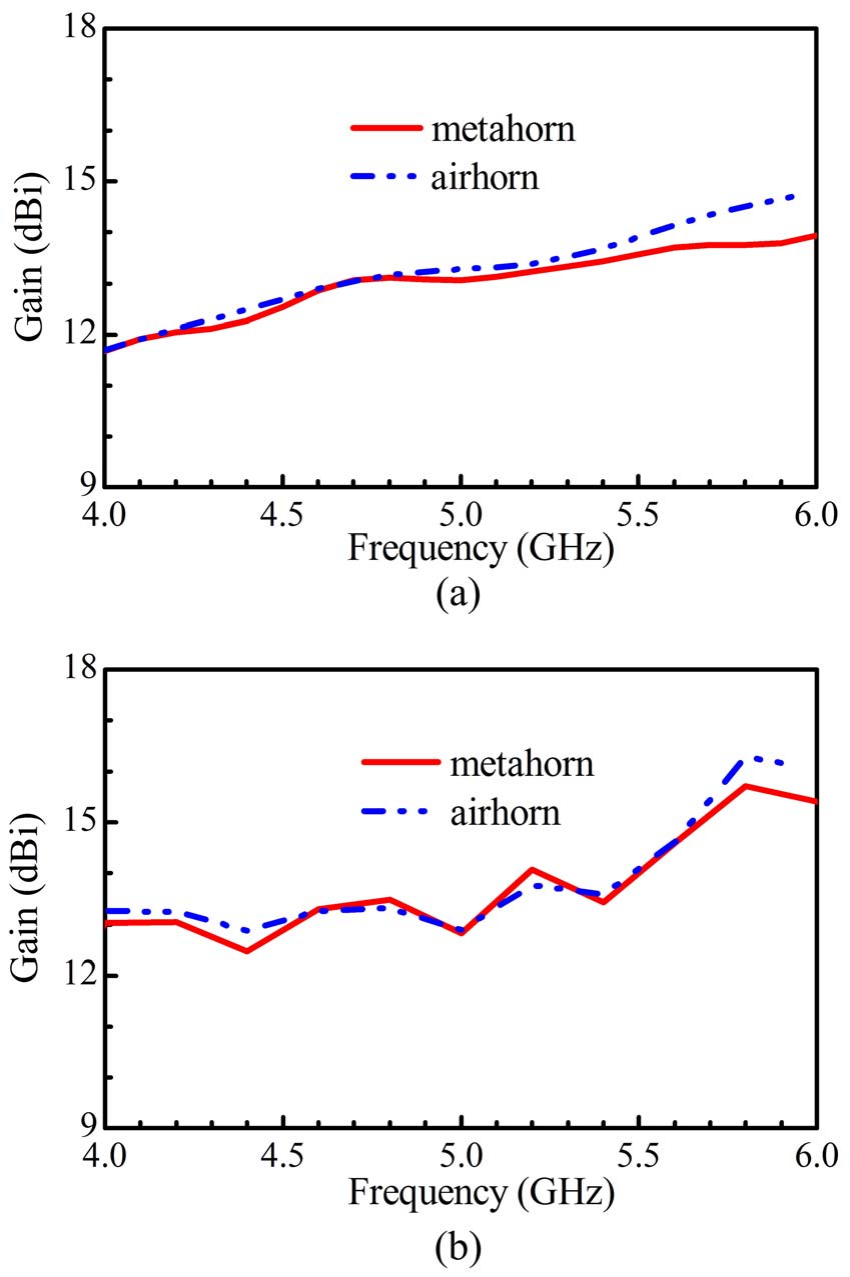
Comparison of gains of the air horn and the meta-horn. (a) Simulated. (b) Measured.

The gains of the air horn and the meta-horn (in the direction of maximum value) are compared in Fig. 7. It is observed that the gain of the meta-horn is slightly lower than that of the air horn. The primary cause is that for the meta-horn the aperture field in E-direction is a tapered one instead of a uniform one.

To verify that the loaded metamaterials do not bring in impedance mismatching for the horn in broadband, the voltage standing wave ratios (VSWRs) for the air horn and the meta-horn are shown in Fig. 5(b) for comparison. According to the results, the meta-horn shows good matching performance in a very broad frequency band. Therefore, the metamaterials are demonstrated to operate in a broadband without mismatching.

In summary, we have presented a method of manipulating the propagation of electromagnetic waves using metamaterials. Aside from controlling the phase of electromagnetic fields, the way of controlling the amplitude is introduced, together with an application to tailor the radiation pattern of a horn antenna without increasing the antenna size. This method can be potentially used to significantly improve the performance of some existing devices from microwave to optical frequencies, and also employed in some adaptive systems.

## Methods

Numerical simulations of the metamaterial-loaded horn antenna were performed by the commercial software, CST Microwave Studio. The material used to fabricate the pyramidal horn was aluminum. The substrate printed with varies length strips was Rogers RO4350B with the relative permittivity 3.66 and loss tangent 0.0037. We used Agilent Vector Network Analyzer to measure the VSWR of the fabricated sample. Far-field radiation pattern was measured in the anechoic chamber using a transmitting horn antenna which is placed 15 meters away from the measured meta-horn antenna and air-horn antenna.

## Author Contributions

M.Q.Q., H.F.M. and T.J.C. conceived the idea. W.X.T. did the theoretical analysis. M.Q.Q. did the simulations and optimization. B.C.P., Z.T. and Y.Z.S. performed the fabrication and measurements. M.Q.Q., W.X.T. and T.J.C. wrote the manuscript based on input from all authors. All authors contributed to the discussions.

## References

[b1] Cui T. J., Smith D. R. & Liu R. Metamaterials: Theory, Design, and Applications (Springer, 2009).

[b2] Schurig D. *et al.* Metamaterial electromagnetic cloak at microwave frequencies. Science 314, 977–980 (2006). 17053110 10.1126/science.1133628

[b3] Liu R. *et al.* Broadband ground-plane cloak. Science 323, 366–369 (2009). 19150842 10.1126/science.1166949

[b4] Valentine J., Li J., Zentgraf T., Bartal G. & Zhang X. An optical cloak made of dielectrics. Nat. Mater. 8, 568–571 (2009). 19404237 10.1038/nmat2461

[b5] Ma H. F. & Cui T. J. Three-dimensional broadband ground-plane cloak made of metamaterials. Nat. Commun. 1, 1–6 (2010). 20975696 10.1038/ncomms1023 PMC2982161

[b6] Landy N. I., Sajuyigbe S., Mock J. J., Smith D. R. & Padilla W. J. Perfect metamaterial absorber. Phys. Rev. Lett. 100, 207402 (2008). 18518577 10.1103/PhysRevLett.100.207402

[b7] Cheng Q., Cui T. J., Jiang W. X. & Cai B. G. An omnidirectional electromagnetic absorber made of metamaterials. New J. Phys. 12, 063006 (2010).

[b8] Smith D. R., Mock J. J., Starr A. F. & Schurig D. Gradient index metamaterials. Phys. Rev. E 71, 036609 (2005). 10.1103/PhysRevE.71.036609 15903607

[b9] Greegor R. B. *et al.* Simulation and testing of a graded negative index of refraction lens. Appl. Phys. Lett. 87, 091114 (2005).

[b10] Ma H. F. *et al.* Experiments on high-performance beam-scanning antennas made of gradient-index metamaterials. Appl. Phys. Lett. 95, 094107 (2009).

[b11] Kundtz N. & Smith D. R. Extreme-angle broadband metamaterial lens. Nat. Mater. 9, 129–132 (2010). 20023631 10.1038/nmat2610

[b12] Ma H. F. & Cui T. J. Three-dimensional broadband and broad-angle transformation-optics lens. Nat. Commun. 1, 124 (2010). 21119637 10.1038/ncomms1126

[b13] Chen X., Ma H. F., Zou X. Y., Jiang W. X. & Cui T. J. Three-dimensional broadband and high-directivity lens antenna made of metamaterials. J. Appl. Phys. 110, 044904 (2011).

[b14] Gu C. *et al.* Experimental realization of a broadband conformal mapping lens for directional emission. Appl. Phys. Lett. 100, 261907 (2012).

[b15] Mei Z. L., Bai J., Niu T. M. & Cui T. J. A half Maxwell fish-eye lens antenna based on gradient-index metamaterials. IEEE Trans. Antennas Propag. 60, 398–401 (2012).

[b16] Ma H. F. *et al.* Three-dimensional gradient-index materials and their applications in microwave lens antennas. IEEE Trans. Antennas Propag. 61, 2561–2569 (2013).

[b17] Qi M. Q., Tang W. X., Xu H.-X., Ma H. F. & Cui T. J. Tailoring radiation patterns in broadband with controllable aperture field using metamaterials. IEEE Trans. Antennas Propag. 61, 5792–5798 (2013).

[b18] Gok G. & Grbic A. Tailoring the phase and power flow of electromagnetic fields. Phys. Rev. Lett. 111, 233904 (2013). 24476273 10.1103/PhysRevLett.111.233904

[b19] Lier E., Werner D. H., Scarborough C. P., Wu Q. & Bossard J. A. An octave-bandwidth negligible-loss radiofrequency metamaterial. Nat. Mater. 10, 252–252 (2011). 10.1038/nmat2950 21278741

[b20] Lier E. Review of soft and hard horn antennas, including metamaterial-based hybrid-mode horns. IEEE Antennas Propag. Mag. 52, 31–39 (2010).

[b21] Ma H. F., Chen X., Yang X. M., Jiang W. X. & Cui T. J. Design of multibeam scanning antennas with high gains and low sidelobes using gradient-index metamaterials. J. Appl. Phys. 107, 014902 (2010).

[b22] Smith D. R., Vier D. C., Koschny T. & Soukoulis C. M. Electromagnetic parameter retrieval from inhomogeneous metamaterials. Phys. Rev. E 71, 036617 (2005). 10.1103/PhysRevE.71.036617 15903615

[b23] Balanis C. A. Antenna Theory: Analysis and Design, 3rd Edition (Wiley-Interscience, 2005).

